# Sir B. Brodic on Calculus

**Published:** 1840

**Authors:** 


### *Sir B. Brodie on Calculus.*

Dr. Linton writes thus to one of the editors of the Maryland Medical and Surgical Journal of Sir Benjamin Brodie and his treatment of stone in the bladder:

Sir Benjamin Brodie is not eloquent; but he indulges little in that which requires it—theory. All that he utters is worthy of the student's note book, and a place in the memory into the bargain. From his appearance he is not more than fifty years of age; his hair is not at all grey, and what is almost a miracle in the profession in England, he is not bald; eyes grey; figure rather slight and elegant, height about five feet nine inches. I have been listening to him on the subject of calculus, and the means of its removal. He asserted that all stones are primarily formed in the kidneys, except those of the phosphate of lime, and those whose nuclei are extraneous substances, as the end of a bougie, &c.; and remarked that in most instances, when the symptoms of calculi are first experienced, in order to pass them it is only necessary to dilate the urethra by bougies, gradually increased in size—then give active diuretics—make the patient retain the bougie as long as possible, until the bladder is full of urine, then let him stand up and withdraw the instrument. The result will be a large stream of water, with which some, if not all the calculi will pass.

When, however, (as is unfortunately too often the case,) the calculi become too large to be passed in this way, he advises the catheter-shaped forceps, which open after introduction into the bladder. He prefers those invented and used by Heurteloup to those of Sir A. Cooper. In fact he regards the former as one of the greatest inventions of modern surgery. When the urethra is sufficiently dilated, these forceps are introduced and opened, and the stone readily falling between their mandibles, is extracted if not too large—if it is the forceps are the very thing to break it, or rather to crush it. Or it may, perhaps, be got as far as the bulb of the urethra, in which situation it is a very safe and simple operation to cut down to it and extract it. In order to perform these operations without injury to the bladder, it should contain at least four ounces of water. Women, owing to the peculiar organization of this part, are more amenable to the operation than the opposite sex, and it is well that such is the fact, for in them, the operation of lithotomy, says Sir B., always produces irremediable incontinence of urine.

When, however, there is stricture of the urethra—enlargement of the prostate, or great iritability of the bladder—and these states of the parts, so unfavorable to the operation just mentioned, cannot be removed, then the knife must be resorted to. Sir B. prefers the lateral operation—I saw him perform it on Friday last. He is a slow but a very safe operator—he regards lithotomy as a very hazardous operation. True, he says, a surgeon will sometimes have the pleasure of seeing some dozens of his patients in succession recovering from it, but he will have better luck than falls to the lot of many, if he does not experience sometimes almost the reverse. He remarked that it was not generally known that the great Cheselden retired from the practice of surgery in consequence of the unfortunate termination of a great many of his operations for stone.—*Md. Jour. of Med. and Sur.*

